# Unfolding political attitudes through the face: facial expressions when reading emotion language of left- and right-wing political leaders

**DOI:** 10.1038/s41598-019-51858-7

**Published:** 2019-10-30

**Authors:** Edita Fino, Michela Menegatti, Alessio Avenanti, Monica Rubini

**Affiliations:** 1 Department of Experimental, Diagnostic and Specialty Medicine (DIMES), Alma Mater Studiorum - University of Bologna, Bologna, Italy; 2 Department of Department of Sociology, Psychology and Education, University Marin Barleti, Tirana, Albania; 3 Department of Psychology, Alma Mater Studiorum University of Bologna, 40126 Bologna, Italy; 4 Centro de Investigación en Neuropsicología y Neurociencias Cognitivas, Universidad Católica del Maule, 3460000 Talca, Chile

**Keywords:** Social behaviour, Neurophysiology

## Abstract

Spontaneous emotionally congruent facial responses (ECFR) to others’ emotional expressions can occur by simply observing others’ faces (i.e., smiling) or by reading emotion related words (i.e., to smile). The goal of the present study was to examine whether language describing political leaders’ emotions affects voters by inducing emotionally congruent facial reactions as a function of readers’ and politicians’ shared political orientation. Participants read sentences describing politicians’ emotional expressions, while their facial muscle activation was measured by means of electromyography (EMG). Results showed that reading sentences describing left and right-wing politicians “smiling” or “frowning” elicits ECFR for ingroup but not outgroup members. Remarkably, ECFR were sensitive to attitudes toward individual leaders beyond the ingroup vs. outgroup political divide. Through integrating behavioral and physiological methods we were able to consistently tap on a ‘favored political leader effect’ thus capturing political attitudes towards an individual politician at a given moment of time, at multiple levels (explicit responses and automatic ECFR) and across political party membership lines. Our findings highlight the role of verbal behavior of politicians in affecting voters’ facial expressions with important implications for social judgment and behavioral outcomes.

## Introduction

Seeing and reading about someone’s smiling or frowning automatically elicits emotionally congruent facial responses (ECFR) in the observer or reader and this effect appears largely unconscious and difficult to suppress^1–4^. For instance, within 500 ms milliseconds after seeing a smiling face there is an activation of the zygomaticus major, the muscle that lifts up the corners of the mouth forming a smile. Similarly, when individuals see a frowning face they react with the activation of the corrugator supercilii, the muscle which draws the eyebrows together forming a frown. Scholars have explained such spontaneous facial congruent activity with the notion of ‘facial mimicry’ and ‘embodied sensorimotor simulation’: when individuals see a facial expression, they construct a simulation of the observed expression in (pre)motor and somatosensory brain networks which would reactivate related concepts, affective states, and autonomic and behavioral changes^5–7^. Embodied simulations could result in emotionally congruent facial muscle activity in the perceiver and contribute to emotion recognition, perspective taking and empathy^8,9^.

Remarkably, a growing body of research has shown that these seemingly automatic processes are also context-dependent and sensibly modulated by social information (for reviews see^10–12^). Indeed, ECFRs are stronger towards members of one’s own social group and reduced or even incongruent towards members of other social groups^13–16^. They are more likely for social targets eliciting positive attitudes^17^ and people who like each other tend to have enhanced ECFR to one another’s expressions, which in turn enhances one’s feelings of liking and affiliation^18–20^. On the contrary, for disliked individuals and those we are in competition with, a complete lack of congruent facial reactions, or even incongruent reactions have been observed^21,22^. This body of evidence can be interpreted in light of neuroscience research showing an early processing of socially and emotionally relevant cues within a few milliseconds of exposure^12,23–25^. Hence, ECFRs likely reflect the late peripheral correlates of an early and dynamic integration of social cues with contextual information that rely on simultaneously top-down and bottom-up processes^23–27^.

Only a handful of studies have examined how political affiliation affects ECFR by measuring facial electromyography (EMG) of participants while watching television clips^28^ or emotion pictures of political leaders^13^. McHugo, Lanzetta, and Bush^28^ found stronger facial simulation of smiles in supporters relative to non-supporters, while anger expressions were not simulated by neither groups of participants. Different results were found by Bourgeois and Hess^13^, who found that participants smiled about equally in response to smiles of both ingroup and outgroup politicians, while they showed enhanced ECFR for anger expressions of the ingroup politician only. In sum, while ECFR is significantly affected by political group membership and, more generally, by the perceiver’s attitudes toward target politician^13,28^, facial reactivity might also depend on the contextual framing of the emotion involved^10–12,26,27^. This explains the inconsistency of findings on facial response to ingroup and outgroup anger since in the intergroup context ECFR to anger might signal an escalation of conflict. Different is the case with smiling, as it is often indiscriminately corresponded for both ingroup and outgroup members, reflecting the fact that mimicry of smiles incurs low or no such social costs and that the meaning of smiles might be contingent on the context^6,12,26,27^.

It should be noted that these studies relied on nonverbal stimuli such as pictures or video excerpts of political leaders’ emotional expressions, and that emotion language of politicians has not received due attention. Embodied cognition research has shown that ECFRs also occur during processing of linguistic stimuli of emotional content. Words referring to emotional concepts or verbs referring to facial expressions and emotional states elicit congruent facial^4,29^. Similarly, people react with congruent facial expressions when reading about emotion expressions or emotion states of third persons^2,30^. However, whether and to what extent facial activation induced by emotional language attributed to political leaders is modulated by shared political orientation of readers and politicians remains unexplored. The present study aims to fill this gap by examining whether reading verbs describing positive and negative emotions of left- and right-wing politicians induces differential facial muscle activity in readers of left and right political orientation.

Participants read and evaluated subject-verb sentences where verbs referring to positive or negative emotions were attributed to either left-or right-wing politicians, such as ‘Renzi smiles’, ‘Berlusconi frowns’. Facial muscle activation was assessed by recording EMG activity from the corrugator supercilii (CS; brow) and zygomaticus major (ZM; cheek) muscles. In line with previous studies on embodied simulations of emotional concepts^2,4,29^, we expected to detect ECFR (Hypothesis 1, *valence effect*), that is a higher activation of the CS (frown) muscle, when participants read sentences composed of negative rather than positive emotion verbs, and a higher activation of the ZM (smile) muscle when participants read sentences composed of positive rather than negative emotion verbs. Remarkably, building on previous research showing an influence of group membership and social context on psychophysiological markers of sensory, emotional and motor reactivity^12,23–27,31^, we predicted an effect of group membership on ECFR to emotion language attributed to political leaders. We formulated two interrelated hypotheses concerning the influence of group membership on ECFR. We predicted that participants would show ECFR to positive and negative emotional verbs attributed to ingroup politicians, whereas no significant difference was expected in response to positive and negative verbs attributed to outgroup politicians (Hypothesis 2, *ingroup selectivity of ECFR*). Specifically, we expected higher activation of CS in response to negative relative to positive emotional verbal expressions of ingroup politicians only. Similarly, we predicted higher activation of the ZM in response to positive compared to negative verbal expressions attributed to ingroup politicians only. Additionally, we hypothesized that emotion language attributed to left-and-right wing politicians would elicit higher ECFR in readers of same compared to opposing political affiliation (Hypothesis 3, *intergroup differences in ECFR*). Specifically, we predicted higher CS activity when participants read negative emotion verbs attributed to ingroup than outgroup politicians and higher ZM activity when participants read positive emotion verbs attributed to ingroup relative to outgroup politicians. For the sake of clarity, these two interrelated hypotheses will be presented separately in the Results section.

## Results

We measured ECFR using facial EMG recording in 28 left-wing and 25 right-wing young voters. Data were collected between January and October 2013, a few months prior to Matteo Renzi’s confirmation at the head of the major left-wing party (end of 2013) and the subsequent unprecedented electoral support he rallied in the Italian elections for the European Parliament (March 2014).

### Behavioral data

#### Manipulation check

All participants in the left-wing group reported being left-wing (1.96 ± 0.62), whereas all participants in the right-wing group reported being right-wing (5.36 ± 0.56), (F(1,52) = 429.59, *p* < 0.001, η^2^ = 0.892). Positive emotion verbs were considered as very positive (5.59 ± 0.70), (*t*(53) = 16.6, *p* < 0.001), and negative emotion verbs were considered as very negative (2.50 ± 0.60), (*t*(53) = −18.2, *p* < 0.001). All participants correctly identified Alfano and Berlusconi as right-wing politicians, and Bersani and Renzi as left-wing politicians. Moreover, Alfano (4.74 ± 1.41), (*t*(53) = 24.9, *p* < 0.001), and Berlusconi (5.46 ± 1.26), (*t*(53) = 31.6, *p* < 0.001), were considered as representing the right-wing, whereas left-wing politicians Bersani (4.83 ± *SD*), (*t*(53) = 25.6, *p* < 0.001), and Renzi (4.51 ± 1.34), (*t*(53) = 24.7, *p* < 0.001), were considered as representative of the left-wing.

#### Political attitudes

Table 1a showed means (SD) scores on political attitudes of left- and right-wing participants for right- and left-wing political leaders. The significant main effect of target politician, (*F*(3, 156) = 30.06, *p* < 0.001, η^2^ = 0.366), showed that Renzi (left-wing) was the most preferred politician (4.40 ± 0.21), followed by Bersani (left-wing; 3.60 ± 0.17), Berlusconi (right-wing; 2.65 ± 0.17), and Alfano (right-wing; 2.31 ± 016), (all *p*s < 0.006). There was a trivial main effect of participants’ political orientation, (*F*(1, 52) = 10.10, *p* = 0.002, η^2^ = 0.163), with right-wing participants reporting higher scores (3.57 ± 0.15) than left-wing participants (2.91 ± 0.14). The interaction was significant, (*F*(3, 156) = 49.59, *p* < 0.001, η^2^ = 0.488). Pairwise comparisons revealed that left-wing participants had more favorable attitudes for left-wing politicians Bersani and Renzi than for right- wing ones Berlusconi, and Alfano, (all *p*s < 0.001). Right-wing participants showed a more favorable attitude for right-wing politicians Berlusconi and Alfano when compared to left-wing politician Bersani, (all *p*s < 0.001) but not when compared to left-wing politician Renzi, (all *p*s > 0.117). Overall, these results showed that Renzi (left-wing) was the most favorite politician among both left- and right-wing participants, (*p* = 0.706), and that, surprisingly, right-wing participants did not differentiate their attitudes towards Renzi and right-wing politicians.

**Table 1:** Means (SD) scores on political attitudes and voting intention of left- and right-wing participants for right- and left-wing political leaders.

a.	Political Attitudes		b.	Voting Intentions	
	Right-wing participants	Left-wing participants		Right-wing participants	Left-wing participants
**Right-wing politicians**					
Berlusconi	4.20 ± 1.74 a	1.10 ± 0.49 c		4.08 ± 0.31 a	1.03 ± 0.29 a
Alfano	3.35 ± 1.69 b	1.27 ± 0.49 c		3.12 ± 0.26 b	1.06 ± 0.24 c
**Right-wing politicians**					
Bersani	2.43 ± 1.08 c	4.78 ± 1.44 b		1.56 ± 0.33 c	5.03 ± 0.31 d
Renzi	4.32 ± 1.74 ab	4.48 ± 1.41 b		3.16 ± 0.39 ab	4.51 ± 0.36 d

Note. Means with different subscripts differ significantly (*p*s < 0.05) within row and column for each panel.

#### Voting intention

Table 1b showed means (SD) scores on voting intention of left- and right-wing participants for right- and left-wing political leaders. The main effect of target politician, (*F*(3, 156) = 13.10, *p* < 0.001, η^2^ = 0.201), showed that participants’ have higher voting intentions towards Renzi (3.83 ± 0.26) than towards Berlusconi (2.55 ± 0.21), *p* = 0.001, and Alfano (2.09 ± 018), (*p* < 0.001), whereas no significant difference was found between Renzi and Bersani (3.29 ± 0.22), (*p* = 0.094). Voting intentions for Bersani were higher than those for Berlusconi, (*p* = 0.024), and Alfano, (*p* < 0.001), who in turned obtained lower scores than Berlusconi, *p* = 0.003. The significant interaction, (*F*(3, 156) = 49.92, *p* < 0.001, η^2^ = 0.490), revealed that left-wing participants were more intentioned to vote for left-wing compared to right-wing politicians, (all *p*s < 0.001). Right-wing participants showed stronger voting inclination for right-wing politicians Berlusconi and Alfano when compared to left-wing politician Bersani, (all *p*s < 0.001), but not when compared to left-wing politician Renzi, (all *ps* > 0.560). Thus, left-wing participants were more intentioned to vote for ingroup politicians, whereas right-wing participants had higher voting intention for right-wing politicians when compared to Bersani, but not when compared to Renzi.

### Physiological data (ECFR): main analysis

#### Corrugator supercilii

**Figure 1:**
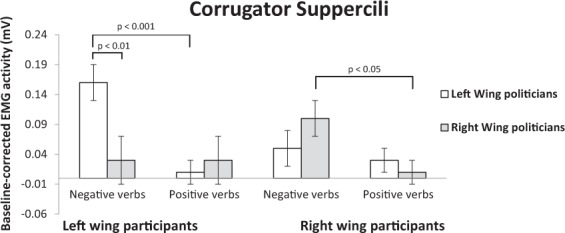
Mean activity levels (expressed in mV) of the corrugator supercilii muscle of left- and right-wing participants in response to positive and negative emotion expressions of left- and right-wing politicians.

The participant political orientation × political party × valence × linguistic category ANOVA on CS data showed a significant main effect of valence (see Fig. 1), thus supporting Hypothesis 1: we observed higher CS activation when participants read sentences referring to negative (0.09 ± 0.02) compared to positive emotion expressions (0.02 ± 0.01), (*F*(1, 52) = 10.32, *p* = 0.002, η^2^ = 0.166.) The participant political orientation × political party interaction was significant, (*F*(1, 52) = 4.71, *p* = 0.035, η^2^ = 0.08), and it was qualified by the significant three way participant political orientation × valence of emotion expression × political party interaction, (*F*(1, 52) = 9.84, *p* = 0.003, η^2^ = 0.16).

In line with the predicted *ingroup selectivity of ECFR* (Hypothesis 2), pairwise comparisons revealed that left-wing participants showed higher CS activity in response to negative (0.16 ± 0.04) than positive (0.01 ± 0.03) emotion expressions of left-wing (ingroup) politicians, (*p* < 0.001). No significant difference was found when left-wing participants read negative (0.03 ± 0.04) and positive (0.03 ± 0.02) emotion expressions of right-wing (outgroup) politicians, (*p* = 0.98). Results of right-wing participants also supported Hypothesis 2. Frowning response to ingroup politicians’ expressions were larger for negative (0.11 ± 0.04) than positive (0.01 ± 0.03) emotion expressions, (*p* = 0.015), whereas no difference was found for response to negative (0.06 ± 0.04) and positive (0.04 ± 0.03) expressions of outgroup politicians, (*p* = 0.60). CS activity of right-wing participants did not show differential activation when reading negative emotion expressions of right-compared to left-wing politicians, (*p* = 0.23). As expected, right-wing participants did not show significant differences in muscular activation when reading positive emotion verbs attributed to ingroup relative to outgroup politicians, (*p* = 0.35). No other significant effect emerged, (all *F*s < 1.41, all *p*s > 0.06).

Hypothesis 3 (*intergroup differences in ECFR*) was supported only for left-wing participants, who frowned more in response to negative emotion expressions of left- (ingroup) relative to right-wing politicians (outgroup), (*p* = 0.002). No significant difference in CS activity was found for positive emotion expressions attributed to ingroup relative to outgroup politicians, (*p* = 0.32).

#### Zygomaticus major

**Figure 2:**
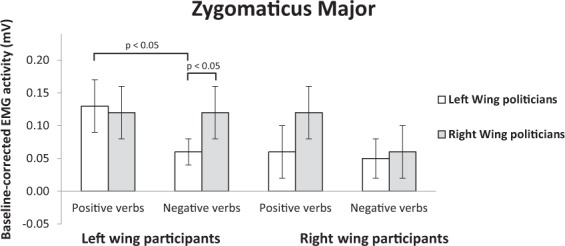
Mean activity levels (expressed in mv) of the zygomaticus major muscle of left- and right-wing participants in response to positive and negative emotion expressions of left- and right-wing politicians.

The participant political orientation × political party × valence × linguistic category ANOVA on ZM data showed a main effect of valence, supporting Hypothesis 1, with higher ZM activation in response to positive (0.11 ± 0.02) than negative emotion expressions (0.07 ± 0.03), (*F*(1, 52) = 4.32, *p* = 0.042, *η*^2^ = 0.08). Although the participant political orientation × valence of emotion expression × political party interaction showed only a trend towards significance, (*F*(1, 52) = 3.135, *p* = 0.082, *η*^2^ = 0.06), we performed pairwise comparisons to directly test our hypotheses (Fig. 2). Results fully supported Hypothesis 2 for left-wing participants, who showed larger ZM activation in response to positive (0.14 ± 0.05) compared to negative (0.06 ± 0.03) emotion expressions of ingroup politicians, (*p* = 0.050), whereas no difference was found between response to positive (0.12 ± 0.05) and negative (0.13 ± 0.05) expressions of outgroup politicians, (*p* = 0.81). Results supported Hypothesis 2 for right-wing participants, as revealed by the greater ZM activation in response to positive (0.12 ± 0.05) vs. negative (0.06 ± 0.05) emotion expressions of ingroup politicians, although the comparison was only marginally significant, (*p* = 0.071). As expected, the difference between positive (0.07 ± 0.05) and negative (0.06 ± 0.03) expressions of outgroup politicians was not significant, (*p* = 0.78).

With regard to Hypothesis 3, left-wing participants did not show significantly different zygomaticus activation when reading positive emotion expressions of ingroup relative to outgroup politicians, (*p* = 0.59). No significant difference emerged for negative emotion verbs attributed to ingroup and outgroup politicians, (*p* = 0.10). Partially confirming results were found for right-wing participants who showed a marginally significant difference in their smile response to positive emotion expressions of ingroup relative to outgroup politicians, (*p* = 0.085). No significant difference emerged between negative verbs attributed to ingroup and outgroup politicians, (*p* = 0.92).

### Physiological data (ECFR): supplementary analysis

The fact that the predicted *intergroup differences in ECFR* (Hypothesis 3) were supported only for left-wing but not right-wing participants was somehow unexpected. Considering the results on political attitudes towards individual politicians and voting intention scores which surprisingly showed a consistently positive evaluation of left-wing leader Renzi among right-wing participants, we reasoned that such favorable attitudes could be reflected in the lack of significant differences in ECFR to ingroup and outgroup politicians among right-wing participants. We thus conducted further participant political orientation × political party × valence × linguistic category ANOVAs in order to explore this possibility. In these analyses left-wing politician Renzi was removed from the left-wing political group. We expected that dissociating participants’ responses to Renzi from the analysis should boost ingroup and outgroup differences in the facial muscle response of right-wing participants.

#### Corrugator supercilii

**Figure 3:**
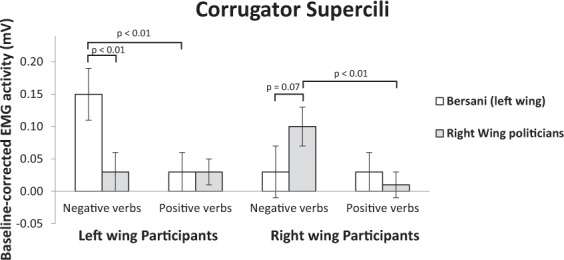
Mean activity levels (expressed in mv) of the corrugator supercilii muscle of left- and right-wing participants in response to positive and negative emotion expressions of Bersani (left-wing) and right-wing politicians.

As shown in Fig. 3, results confirmed the main effect of valence of emotion expression, indicating that participants showed significantly higher activation of the CS when reading verbs referring to negative (0.12 ± 0.03) than positive emotion expressions (0.01 ± 0.02), (*F*(1, 52) = 5.57, *p* = 0.022, η^2^ = 0.10). As expected, the participant political orientation × valence of emotion expression × political party interaction was significant, (*F*(1, 52) = 9.93, *p* = 0.003, η^2^ = 0.16). In line with Hypothesis 2, left-wing participants displayed higher CS activity when reading negative (0.16 ± 0.05) vs. positive (0.04 ± 0.03) emotion verbs attributed to the ingroup politician, (*p* = 0.006). The same effect was found for right-wing participants, who displayed greater frowning when reading negative (0.11 ± 0.04) than positive (0.01 ± 0.03) emotion verbs expressed by ingroup politicians, (*p* = 0.015). No such effect was observed when participants read emotion verbs attributed to outgroup politicians. In that case, both left- and right-wing participants did not show differential activation of CS in response to positive and negative emotion expressions of right- (negative: 0.04 ± 0.04; positive: 0.03 ± 0.03, *p* = 0.99) and left-wing politicians respectively (negative: 0.03 ± 0.03; positive: 0.03 ± 0.05;*p* = 0.92).

In line with Hypothesis 3, the CS activation of left-wing participants was higher when reading negative emotion expressions of the ingroup politician compared to outgroup politicians, (*p* = 0.004). The same effect was found for right-wing participants, although the difference was only marginally significant, (*p* = 0.078). No significant difference in CS activity was found for positive verbs attributed to ingroup and outgroup politicians for both left, (*p* = 0.52), and right-wing participants, (*p* = 0.93). See Supplementary results, S2 for further analysis comparing participants’ facial reactions to Renzi with those of other right-wing politicians by dissociating the data of the other left-wing politician, Bersani.

#### Zygomaticus major

**Figure 4:**
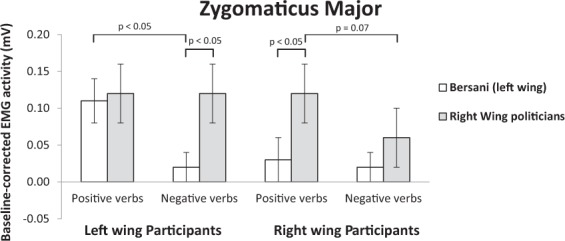
Mean activity levels (expressed in mv) of the zygomaticus major muscle of left- and right-wing participants in response to positive and negative emotion expressions of Bersani (left-wing) and right-wing politicians.

As shown in Fig. 4, results of the same ANOVA on ZM data confirmed the main effect of valence of emotion expression, with participants showing higher ZM activation in response to positive (0.09 ± 0.02) than negative emotion verbs (0.06 ± 0.02), (*F*(1, 52) = 4.67, *p* = 0.035, η^2^ = 0.080). The main effect of linguistic category indicated that ZM was activated more when participants read state verbs (0.10 ± 0.03) than action verbs (0.05 ± 0.01), (*F*(1, 52) = 4.96, *p* = 0.030, η^2^ = 0.09). The main effect of political group showed a greater smiling response when emotion verbs were attributed to right-wing politicians (0.10 ± 0.03) than to the left-wing politician (0.05 ± 0.01), (*F*(1, 52) = 6.40, *p* = 0.014, η^2^ = 0.110).

The participant political orientation × valence of emotion expression × political group interaction was significant, (*F*(1, 52) = 5.46, *p* = 0.023, η^2^ = 0.095). In line with Hypothesis 2, when left-wing participants read emotion verbs attributed to the ingroup politician, their ZM activity was higher in response to positive (0.11 ± 0.03) than negative (0.03 ± 0.02) emotion verbs, (*p* = 0.011). However, their ZM response did not show difference for positive emotion verbs attributed to the ingroup politician than outgroup politicians (0.12 ± 0.05), (*p* = 0.815). As expected, no significant difference was observed when left-wing participants read positive and negative (0.13 ± 0.05) emotion verbs attributed to outgroup politicians, (*p* = 0.81). Furthermore, left-wing participants displayed an incongruent facial reaction as they smiled more when reading negative emotion verbs attributed to outgroup politicians than the left-wing politician, (*p* = 0.013).

The ZM response of right-wing participants was higher when reading positive (0.12 ± 0.05) than negative (0.06 ± 0.05) emotion verbs of ingroup politicians, although the difference was marginally significant, (*p* = 0.071). No difference was found between positive (0.04 ± 0.03) and negative (0.03 ± 0.02) emotion verbs attributed to outgroup politicians (*p* = 0.73). As expected, right-wing participants showed greater activation of the ZM when reading positive emotion verbs attributed to ingroup relative to outgroup politicians, (*p* = 0.028). No significant difference in the zygomaticus response was found for negative emotion verbs attributed to ingroup vs. outgroup politicians, (*p* = 0.37).

Overall, the results obtained when the responses to Renzi were removed from the left-wing political group fully confirmed Hypothesis 2 for both left- and right-wing participants, who displayed congruent facial reactions in response to ingroup politicians only. Results of right-wing participants also supported Hypothesis 3 by showing that their facial responses to emotional verbs of politicians belonging to the left and right political orientation were different. Conversely, left-wing participants, as expected from this specific analysis, showed a similar activation of the smile muscle in response to positive expression verbs of target politicians independent of their political orientation. Interestingly, though, they also smiled in response to negative emotion expressions of right-wing politicians, which is clearly an incongruent facial reaction notably linked to a counter-empathic response^14,21^.

## General Discussion

The present study uniquely highlighted ECFR to emotion language of political leaders and a modulating role of political orientation and attitudes towards target politicians. Importantly, through the integration of behavioral and physiological measures we were able to capture political attitudes consistently across different measures and at varying degrees of awareness. By measuring automatic facial muscle activation, we found that reading verbal stimuli that describe political leaders’ emotions produces ECFR in the readers, such that positive expressions elicit “smiles” whereas negative expressions elicit “frowns” (Hypothesis 1). Remarkably, our findings highlighted a strong *ingroup selectivity of ECFR* (Hypothesis 2), as reading about politicians’ positive and negative emotions yielded ECFR in readers for ingroup but not for outgroup politicians. Furthermore, we found *intergroup differences in ECFR* (Hypothesis 3) for left-wing subject, who showed a greater frowning response when reading negative emotion expressions of politicians belonging to the left compared to the right political orientation. Conversely, on a first analysis, right-wing participants showed an undifferentiated smiling and frowning response to positive and negative emotion expressions of right- and left-wing politicians. Taken together, our findings support the idea that ECFRs are context-dependent and sensibly modulated by social information^23–27^. However, to better grasp the meaning of these results, we considered findings from explicit measures on political attitudes and voting intention, which consistently indicated that right-wing participants held favorable attitudes towards left-wing politician Renzi despite they clearly identified him as a left-wing political leader. So much so that when it came to their political attitudes and inclination to vote, they did not differentiate between ingroup (right-wing) politicians and left- wing leader Renzi. On this basis, we reasoned that favorable attitudes towards Renzi among right-wing participants could have obscured differences in their facial response to ingroup and outgroup politicians’ expressions. Supplementary analysis where we compared facial reactions to emotion language of right- and left-wing politicians by dissociating Renzi from the left-wing group revealed that language-based ECFR of right-wing participants were indeed enhanced for emotion expressions of ingroup but not outgroup politicians. Most likely, positive attitudes towards Renzi concealed the hypothesized intergroup differences in facial simulation of right-wing participants. To put it differently, our findings revealed a “favorite political leader effect” according to which attitudes toward an individual political leader, more than mere left vs. right political orientation, affected embodied simulation of emotion language.

The present study builds upon and expands previous research showing that psychophysiological markers of sensory, emotional and motor reactivity are sensitive to social influences^12,23–27,31^. Our findings support the view that integration of contextual and social information occurs very rapidly^12,23–27,31^ and affects the magnitude of a relatively late phenomenon like ECFRs to linguistic stimuli. Moreover, our study complements applied research on the social moderators of ECFR, political attitudes and voting behavior. First, we highlighted that voters’ ECFR to language describing politicians’ emotion expressions could signal political attitudes that go beyond explicitly expressed political orientation. Our results revealed that political attitudes sensibly modulated ECFRs, as shown by the fact that political leaders singularly favored by left- and right-wing electorates triggered similar facial effects. Renzi’s appeal as a political leader throughout 2013 was steadily growing thanks to his ability to connect to voters beyond the party divide^32–35^. Our data were collected only a couple of months prior to his confirmation at the head of the major left-wing party (end of 2013) and the subsequent unprecedented electoral support (more than 40%, the best result ever reached by his political party) he rallied in the Italian elections for the European Parliament (March, 2014). By measuring automatic ECFR, we could therefore gauge on the general tide of positive attitudes that was actually building up in favor of Renzi, who emerged in that particular moment and in that particular context as *the* political leader able to move audiences beyond the logic of left and right, building a wide consensus that flowed into actual electoral support.

Moreover, we showed for the first time that language describing politicians’ emotions and the political context in which such stimuli are embedded affect spontaneous subtle non-verbal behaviors such as ECFR^10^. Going beyond previous evidence on the social and political factors affecting ECFR^11^, we clearly found facial sensitivity to political leaders’ emotion expressions even when such information is conveyed through linguistic material: what people read about politicians, being in newspaper or in social media, has the power to affect their facial reactions the same as when viewing politicians’ happy or angry faces. This can have important implications for eliciting congruent social judgments^4,36^ and for behavioral outcomes especially as they relate with voting behavior. Recent research has shown that automated facial expressions analysis could be used to predict with good accuracy voting behaviors^37^. Our findings suggest that ECFR to written media and reports on candidates’ emotional behaviors could also serve as indices of embodied political attitudes with potential implications for predicting voting decisions^38^.

One potential limit of the present study is the use of a self-reported one-dimensional scale of political orientation. There is a considerable debate on the validity of such a measure to capture the political ideology of electorates (for a review, see^39^). Nevertheless, it should be noticed that the traditional self-identification measure on a single scale performs quite well as a predictor of political behavior (e.g., in predicting the vote^33,40,41^). Furthermore, a great body of research has demonstrated the utility of unidimensional ideological axes (left – right) as the main political orientation instrument used by individuals to navigate in the Italian political context, at least until the moment of data collection^34,42^. Thus, we could consider such a measure as valid for the purpose of the present study. Future research should consider whether our evidence could be confirmed using more complex measures of political orientation and ideology.

Another potential limit of the present study is related to the fact that we could not provide a clear-cut explanation of why left-wing participants did not differentiate their smile response to positive expressions of ingroup and outgroup politicians. This result is in line with studies on the effects of group membership on facial mimicry^13,15^, suggesting that smiling in response to expressions of opponents incurs low social costs in the political intergroup context. Interestingly, we found an incongruent facial activation pattern instead, that is smiles in response to negative expressions of outgroup politicians. Given that ZM activation is also observed when individuals report feeling negative emotions, it is possible that such a pattern reflects an instance of ‘schadenfreude’ or counter empathic response^21,43^ probably due to morally tainted attitudes of left-wing participants towards particularly controversial Italian right-wing politician Berlusconi, who is especially despised among Italian left-wing voters^44^. This explanation is in line with the higher liking of left-wing participants of negative verbal expressions of outgroup politicians (see Supplementary results, S1). Future studies should test this possibility and also examine whether participants’ smiles express genuine enjoyment or represent a reaction related with negative emotions by measuring the simultaneous activation of the ZM and the orbicularis oculi (OO) muscle, which is supposed to be activated only when a smile is associated with truly felt enjoyment^6,45^.

Lastly, our sample was not balanced in terms of gender and we cannot exclude that gender may have had an impact on language based ECFR. It should be noted however that previous facial EMG research^42^ has not evidenced significant differences in terms of gender (e.g., for instance, it has been found that levels of facial muscle activation are more pronounced in females compare to males, but no significant differences are reported in terms of facial pattern congruency between male and female subjects). Future research should investigate language triggered ECFR using a more gender balanced sample.

## Conclusion

This research is the first to show that reading about emotion expressions of ingroup political leaders elicits greater ECFR than reading about expressions of outgroup politicians. We also showed that, beyond shared political orientation, such facial reactivity reflects political attitudes toward individual leaders which could flow into actual electoral support. Going beyond previous research, and through the integration of behavioral and psychophysiological methods we were able to consistently tap on a ‘favored political leader effect’ whereby political attitudes towards an individual politician were sensibly captured at a given moment of time, at multiple levels of awareness (explicit responses and automatic facial reactions) and across political party membership lines. ECFR to language describing politicians’ emotions proved a highly sensitive and informative measure, as it was able to reveal, at a given time the political attitudes and voting intentions of the electorate. It should also be noted that the effect we found was closely related to a well-defined moment in time, depicting the glory of a political leader: *sic transit gloria mundi*, very likely so do ECFRs toward formerly glorious leaders.

## Method

### Participants

Fifty-four (43 females; 22 ± 1.42) undergraduate Italian students of the University of Bologna participated in the experiment for academic credits. Of these, 28 reported being of left-wing and 25 of right-wing political orientation. Participants were selected on the basis of a pre-test in which we asked 170 students to indicate their political orientation with the following item “In politics people talk of *left* and *right*. Using this card, where would you place yourself on this scale, where 1 indicates the left, 4 indicates center and 7 indicates the right?” (see^33,46^). This item, is routinely administered in multinational surveys (e.g., European Social Survey and European Values Survey), is as a valid and reliable measure of left–right political orientation^40,46^ and has been the basis of several political psychological studies in the Italian context^35,42,47^. According to recent studies, self-placement on a left – right dimension has been the primary criterium utilized by the Italian electorate to perceive, categorize, and evaluate candidates, parties and issues in political terms^34,42,48^. Participants who reported clear-cut political attitudes in the pre-test (N = 62), that is those who ticked below ‘3’ (left-wing political orientation), and those above ‘5’ (right-wing political orientation) were solicited to take part in the study. Among them, 54 (87%) agreed to participate. Prior to the start of the experiment, they read the ethical approval statement and filled out the informed consent form. Data were collected between January and October 2013.

### Linguistic stimuli

The stimulus material consisted in 12 subject-verb sentences where the subject was either a left-or right-wing Italian politician and the verb referred to either positive or negative emotion expressions (e.g., ‘Renzi smiles’, ‘Berlusconi frowns’). Politicians were selected through a pretest in which 30 undergraduate students were asked to write down the names of the first right- and left-wing national politicians who came to their mind. Four politician names were selected on the basis of the highest frequencies of citation. Bersani (24 citations) and Renzi (21 citations) who belonged to the main Italian left-wing political party (Democratic Party), and Berlusconi (26 citations) and Alfano (23 citations), belonging to the main right-wing political party (People of Freedom) and representing the political opposition at the time. It should be noted that both parties were born and defined as moderate (or center-left and center-right) parties, so much so that more extreme left and right parties do exist in the Italian context. The verbs used in the sentences were derived from Fino *et al*.^2^ and matched for word length and frequency in language use (see^2^). Half of them were action verbs and directly refer to the action of smiling or frowning, and the other half were state verbs, referring to more abstract emotional states^49^ (see Supplementary material).

### Procedure and measures

Participants were asked to participate in a study on language of politics. In order to conceal the real purpose of the study they were told that skin conductance levels would be recorded via sensors placed on the face during a reading task. The computer task consisted in sequential presentation of verbal material on a monitor and subsequent evaluation of stimulus likeability by right hand clicking on a 5-point scale (1 = *I don’t like it at all;* 5 = *I like it very much*). The scale appeared on the monitor after each stimulus sentence and disappeared upon response (see Supplementary results S1 for results on liking of politicians’ linguistic expressions).

After the computer task, participants completed a questionnaire including manipulation checks, political attitudes and voting intention. They were first asked to indicate their political orientation on the same item used in the pre-test. As manipulation checks, participants rated the valence of each stimulus sentence (1 = *very positive*; 7 = *very negative*), indicated the political affiliation of each politician by choosing between two options (left or right), and rated how much each target politician represented the political wing they had indicated in the previous question (1 = *not at all;* 7 = *very much*). Then, we assessed participants attitudes towards target politicians by means of a series of items aimed to tap into the affective and cognitive components of attitudes which have been shown to operate as partially distinct determinants of political candidate evaluation and subsequent voting behavior^50,51^. Items referred to the cognitive and affective components of participants’ political attitudes were measured by asking “How much does target politician represent your political ideas?”, “How much do you like target politician?”, “Would you be happy if target politician won in the coming elections?”. Given the strong correlations between the answers (Bersani’s α 0.87; Berlusconi’s α = 0.94; Alfano’s α = 0.91; Renzi’s α = 0.82), they were averaged into four variables representing participants attitudes toward each politician. The behavioral component of the attitudes was measured by asking participants on their voting intention for each target politician in the coming election (1 = *not at all*; 7 = *very much*). When asked about their ideas regarding the purpose of the experiment, no participant revealed awareness of the hypotheses, and none suspected that facial muscular activation was being measured. All participants reported normal or corrected-to-normal vision.

### Data acquisition and preprocessing

Facial muscle activity was measured using miniature Ag/AgCl (10/4 mm) surface electrodes attached over the left CS (brow) and ZM (cheek) muscle regions according to Fridlund and Cacioppo’s guidelines^52^. The EMG raw signal was measured with a Biopack MP36 (Systems Inc.), was pass filteredn online with a 20–250 Hz passband and a 50 Hz notch filter and was full-wave rectified offline. Stimuli were presented in a random order with E-prime software (Psychology Software Tools, Pittsburgh, PA) on a monitor located approximately 1 m from the participant. Each trial was composed of a central fixation cross (duration 2000 ms), followed by the presentation of the target stimulus (3000 ms), an evaluation scale (average duration ~4 seconds) and blank screen (1000 ms). The inter-stimulus interval was ~10 seconds allowing sufficient time for facial muscles to relax after stimulus presentation. For each participant three blocks of stimuli were presented with each block containing all the 12 emotion expression verbs and 6 neutral verbs attributed to all politicians in random order, resulting in 72 stimuli presentations altogether. In order to familiarize with the task, participants completed 10 practice trials at the beginning of the experiment.

EMG values in millivolt (mV) were expressed as difference in activity from the pre-stimulus level, defined as baseline (for a similar procedure see^13,17,53^). For each trial and muscle, baseline EMG level was calculated as the mean signal computed during the 1000 ms preceding the sentence stimulus. Then, facial EMG response to target stimuli was computed as the mean EMG signal for 3000 ms of stimulus exposure, which was then baseline corrected. Before statistical analysis, EMG data were collapsed over the 18 trials with the same emotional expression and linguistic category for left-and right-wing political party.

### Data analysis and statistics

All statistical analyses were conducted using SPSS software (version 21.0). Group comparisons were performed through conventional t tests (or Wilcoxon Rank Sum for non-normal distributions) and repeated measures analysis of variance (ANOVA). We carried out 2 (participant political orientation: left-wing, right-wing) × 2 (political party: left-wing, right-wing) × 2 (valence of emotion expression: positive, negative) × 2 (linguistic category: action verbs, state verbs) ANOVAs with repeated measures on the last three factors on EMG data for the CS and ZM muscles separately. The Greenhouse–Geisser correction for degrees of freedom was applied when the sphericity assumption was violated. For cases where repeated measures ANOVA revealed a significant interaction effect, Bonferroni post hoc analyses were used to examine the specific comparisons and significance levels.

### Ethical approval

All participants signed written informed consent. The experiment was conducted in accordance with relevant guidelines and regulations. The study protocol was approved by the Institutional Review Board of University of Bologna.

## Supplementary information

Supplementary Material

Dataset 1

## Data Availability

The datasets generated during and/or analyzed during the current study are available in Microsoft Excel format (Supplementary Dataset 1).
