# Author Correction: Unfolding political attitudes through the face: facial expressions when reading emotion language of left- and right-wing political leaders

**DOI:** 10.1038/s41598-020-58944-1

**Published:** 2020-02-05

**Authors:** Edita Fino, Michela Menegatti, Alessio Avenanti, Monica Rubini

**Affiliations:** 1 Department of Experimental, Diagnostic and Specialty Medicine (DIMES), Alma Mater Studiorum - University of Bologna, Bologna, Italy; 2 Department of Department of Sociology, Psychology and Education, University Marin Barleti, Tirana, Albania; 3 Department of Psychology, Alma Mater Studiorum University of Bologna, 40126 Bologna, Italy; 4 Centro de Investigación en Neuropsicología y Neurociencias Cognitivas, Universidad Católica del Maule, 3460000 Talca, Chile

Correction to: *Scientific Reports* 10.1038/s41598-019-51858-7, published online 30 October 2019

The original version of this Article contained an error in Affiliation 3, which was incorrectly given as ‘Department of Psychology, Alma Mater Studiorum University of Bologna, Bologna, Italy’. The correct affiliation is listed below.

Department of Psychology, Alma Mater Studiorum University of Bologna, 40126, Bologna, Italy

Additionally, Alessio Avenanti was incorrectly affiliated with ‘IRCCS Fondazione Santa Lucia, Roma, Italy’. The correct affiliations are listed below.

Department of Psychology, Alma Mater Studiorum University of Bologna, 40126, Bologna, Italy

Centro de Investigación en Neuropsicología y Neurociencias Cognitivas, Universidad Católica del Maule, 3460000, Talca, Chile

These errors have now been corrected in the PDF and HTML versions of the Article.

