# Supplementary material for: Unfolding political attitudes through the face: facial expressions when reading emotion language of left- and right-wing political leaders
Source: Sci Rep. 2019 Oct 30;9:15689. doi: 10.1038/s41598-019-51858-7 (PMC6821753; doi:10.1038/s41598-019-51858-7)
Supplement: Supplementary file 1 — Supplementary Material [file 41598_2019_51858_MOESM1_ESM.docx]

**Unfolding political attitudes through the face: facial expressions when reading emotion language of left- and right-wing political leaders**

Edita Fino^1,2^ PhD, Michela Menegatti^3^ PhD, Alessio Avenanti ^3,4^ PhD and Monica Rubini^3^ PhD

1 Department of Experimental, Diagnostic and Specialty Medicine (DIMES),

Alma Mater Studiorum - University of Bologna, 40126, Bologna, Italy.

2 Department of Department of Sociology, Psychology and Education, University Marin Barleti, 1000, Tirana, Albania.

3 Department of Psychology, Alma Mater Studiorum University of Bologna, 40126, Bologna, Italy.

4 Centro de Investigación en Neuropsicología y Neurociencias Cognitivas, Universidad Católica del Maule, 3460000, Talca, Chile.

* Corresponding author:

Edita Fino, PhD

Department of Experimental, Diagnostic and Specialty Medicine (DIMES), Alma Mater Studiorum - University of Bologna, Viale Berti Pichat 5, 40126, Bologna, Italy;

Telephone: + 39 051 2091330; E-mail: [edita.fino@unibo.it](mailto:edita.fino@unibo.it)

**Supplementary Results: S1**

**Liking of politicians’ linguistic expressions**

Liking ratings of the verbal stimuli were analyzed with a 2 (participant political orientation) × 2 (valence of emotion expression: positive, negative) × 4 (target politician) ANOVA with repeated measures. Results revealed the main effects of target politician, (*F*(3, 50) = 7.38, *p* < 0.001, η^2^ = .124), and valence of emotion expression, (*F*(1, 52) = 6.16, *p* = 0.016, η^2^ = .106). Participants expressed more liking of emotion expressions of Renzi (2.89 ± 0.06), followed by those of Bersani (2.75 ± 0.06), (*p* = 0.030), Berlusconi (2.66 ± 0.08), (*p* = 0.022), and Alfano (2.50 ± 0.07), (*p* < 0.001). Linguistic expressions of Bersani were liked more than those of Alfano, *p* = 0.013, but no difference was found between expressions of Bersani and Berlusconi, (*p* = 0.412). Expressions of Berlusconi were liked more than those of Alfano, *p* = 0.006. Positive linguistic expressions were liked more (2.84 ± 0.08) than negative ones (2.56 ± 0.07). The target politician × participant political orientation, (*F*(3, 50) = 26.10, *p* < 0.001, η^2^ = .334), and the target politician × valence of emotion expression interactions, (*F*(3, 50) = 8.10, *p* < 0.001, η^2^ = .135), were significant and qualified by the significant three-way interaction, (*F*(3, 50) = 25.67, *p* < 0.001, η^2^ = .331). As shown in Table S1, left-wing participants liked more positive emotion expressions of left-wing politicians Bersani and Renzi compared to right-wing politicians Berlusconi and Alfano, all *p*s < 0.001, whereas no differences emerged between the two left-wing, (*p* = 0.121), and the two right-wing politicians, (*p* = 0.442). Left-wing participants liked more negative expressions of Berlusconi than those of Renzi, (*p* = 0.050), whereas the other comparisons for negative expressions were not significant, (all *p*s > .126). Left-wing participants liked more positive than negative emotion expressions of ingroup politicians, (all *p*s < 0.001), and negative than positive emotion expressions of outgroup politicians, (all *p*s < 0.002).

Right-wing participants liked more positive expressions of Berlusconi than those of Alfano, and Bersani, (all *p*s < 0.001), whose expressions were less liked than those of Alfano, (*p* = 0.007), and Renzi, (*p* < 0.001). No difference was found in liking of positive expressions of Renzi and right-wing politicians, (all *p*s > 0.107). Right-wing participants did not report different liking of negative expressions of politicians, (all *p*s > 0.593). Right-wing participants liked more positive than negative emotion expressions of Berlusconi, (*p* < 0.001), Alfano, *(p* = 0.053), and Renzi, (*p* = 0.021), whereas no difference was found for negative compared to positive emotion expressions of Bersani, (*p =* 0.287). Comparisons between left- and right-wing participants showed that positive expressions of Berlusconi and Alfano were liked more by right- than left-wing participants, (all *p*s < 0.001), whereas those of Bersani were liked more by left- than right-wing participants, (p < 0.001). No difference was found between left- and right-wing participants in liking of positive expressions of Renzi, (*p* = 0.134), and for negative expressions of all politicians, (all *p*s > 0.147).

*Table S1*. Means (SD) of left- and right-wing participants’ liking towards positive and negative verbal expressions of right- and left-wing political leaders.

|  | Positive expressions | | |  | Negative expressions | | |
| --- | --- | --- | --- | --- | --- | --- | --- |
|  | Right-wing participants |  | Left-wing participants |  | Right-wing participants |  | Left-wing participants |
| Right-wing politicians |  |  |  |  |  |  |  |
| Berlusconi | 3.58 ± 0.19 a |  | 1.72 ± 0.18 c |  | 2.48 ± 0.19 a |  | 2.87 ± 0.18 a |
| Alfano | 2.92 ± 0.16 b |  | 1.94 ± 0.15 c |  | 2.44 ± 0.16 b |  | 2.69 ± 0.15 ab |
| Left-wing politicians |  |  |  |  |  |  |  |
| Bersani | 2.28 ± 0.16 c |  | 3.66 ± 0.15a |  | 2.54 ± 0.14 c |  | 2.51 ± 0.13 abc |
| Renzi | 3.13 ± 0.19 abd |  | 3.52 ± 0.17 a |  | 2.46 ± 0.13 d |  | 2.45 ± 0.12 bd |

*Note*. Means with different subscripts differ significantly (*p*s < 0.05) within row and column for each panel.

**Supplementary results: S2**

**Examining right-and left-wing differences in facial reactions**

We singularly confronted participants’ facial reactions to Renzi with those of other right-wing politicians by dissociating Bersani's data (i.e., the other left-wing politician). Given the generalized positive attitudes towards Renzi, we should not find significant difference in right-wing participants’ responses to Renzi compared to right-wing politicians Berlusconi and Alfano. A 2 (participant political orientation) × 2 (political party) × 2 (emotion expression valence) × 2 (linguistic category) ANOVAs, on the CS site without Bersani’s data was performed. Results showed the main effect of emotion expression, with higher CS activation when participants read verbs referring to negative emotion expressions (0.99 ± 0.02) compared to verbs referring to positive emotion expressions (0.01 ± 0.01), (*F*(1, 53) = 10.8, *p* = 0.002, η2 = .17). The participant political orientation × valence × political group interaction was significant, (*F*(1, 53) = 5.60, *p* = 0.022, η2 = .10). Left-wing participants frowned to a larger extent in response to negative emotion expressions of Renzi (0.16 ± 0.03) compared to right-wing politicians (0.03 ± 0.03), (p = 0.007). Right-wing participants did not show differential frowning in response to negative emotion expressions of Renzi (0.08 ± 0.03) and right-wing politicians (0.10 ± 0.03), (p = 0.67). No other significant effect was found, (all *F*s < 1.41), (all *p*s > 0.05). Thus, when responses to Renzi’s linguistic expressions were compared to those of both right-wing politicians Hypothesis 3 was supported for left-wing participants, who differentiated their response to ingroup and outgroup members. However, as expected from this analysis, right-wing participants frowned about equally when reading about negative emotion expressions of ingroup politicians and left-wing politician Renzi.

**Supplementary results: S3 Stimulus Material**

|  | Italian | English |
| --- | --- | --- |
| **Positive Verbs** | | |
| DAV | ridere | to laugh |
|  | sorridere | to smile |
|  | ridacchiare | to grin |
| SV | entusiasmarsi | to be enthused |
|  | gioire | to enjoy |
|  | rallegrarsi | to be excited |
| **Negative Verbs** | | |
| DAV | aggrottare | to scowl |
|  | corrucciare | to frown |
|  | accigliarsi | to glare |
| SV | arrabbiarsi | to get angry |
|  | irritarsi | to get irritated |
|  | infuriarsi | to get furious |
| **Neutral Fillers** | | |
| DAV | passeggiare | to walk |
|  | viaggiare | to travel |
|  | lavorare | to work |
| IAV | cambiare | to change |
|  | cercare | to search |
|  | presentarsi | to present |

Stimulus material consisted in 12 Italian verbs: 6 descriptive action verbs (DAV), 6 state verbs (SV) related to positive and negative emotion expressions, and 6 neutral fillers for which we used descriptive action verbs (DAV) and interpretative action verbs (IAV). Verbs were embedded into subject-verb sentences and were presented to participants in the present tense and attributed to a left- or right-wing politician (e.g., “Renzi smiles”).

**Supplementary Results: S4 Whisker plots**

**Legends**

**Figure.1** Whisker plot of the EMG activity data (expressed in mV) of the corrugator supercilii muscle of left- and right-wing participants in response to positive and negative emotion expressions of left- and right-wing politicians. The lower and upper hinges correspond to the first and third quartiles (the 25th and 75th percentiles). The upper whisker extends from the hinge to the largest value no further than 1.5 * IQR from the hinge (where IQR is the inter-quartile range, or distance between the first and third quartiles). The lower whisker extends from the hinge to the smallest value at most 1.5 * IQR of the hinge. Data beyond the end of the whiskers are plotted "outlying" points and are plotted individually. The diagram also shows the median and distribution of data.

**Figure.2** Whisker plot of the EMG activity data (expressed in mv) of the zygomaticus major muscle of left- and right-wing participants in response to positive and negative emotion expressions of left- and right-wing politicians. The lower and upper hinges correspond to the first and third quartiles (the 25th and 75th percentiles). The upper whisker extends from the hinge to the largest value no further than 1.5 * IQR from the hinge (where IQR is the inter-quartile range, or distance between the first and third quartiles). The lower whisker extends from the hinge to the smallest value at most 1.5 * IQR of the hinge. Data beyond the end of the whiskers are plotted "outlying" points and are plotted individually. The diagram also shows the median and distribution of data.

**Figure.3** Whisker plot of the EMG activity data (expressed in mv) of the corrugator supercilii muscle of left- and right-wing participants in response to positive and negative emotion expressions of Bersani (left-wing) and right-wing politicians. The lower and upper hinges correspond to the first and third quartiles (the 25th and 75th percentiles). The upper whisker extends from the hinge to the largest value no further than 1.5 * IQR from the hinge (where IQR is the inter-quartile range, or distance between the first and third quartiles). The lower whisker extends from the hinge to the smallest value at most 1.5 * IQR of the hinge. Data beyond the end of the whiskers are plotted "outlying" points and are plotted individually. The diagram also shows the median and distribution of data.

**Figure.4** Whisker plot of the EMG activity data (expressed in mv) of the zygomaticus major muscle of left- and right-wing participants in response to positive and negative emotion expressions of Bersani (left-wing) and right-wing politicians. The lower and upper hinges correspond to the first and third quartiles (the 25th and 75th percentiles). The upper whisker extends from the hinge to the largest value no further than 1.5 * IQR from the hinge (where IQR is the inter-quartile range, or distance between the first and third quartiles). The lower whisker extends from the hinge to the smallest value at most 1.5 * IQR of the hinge. Data beyond the end of the whiskers are plotted "outlying" points and are plotted individually. The diagram also shows the median and distribution of data.
